# Characterization of Genetic Basis on Synergistic Interactions between Root Architecture and Biological Nitrogen Fixation in Soybean

**DOI:** 10.3389/fpls.2017.01466

**Published:** 2017-08-23

**Authors:** Yongqing Yang, Qingsong Zhao, Xinxin Li, Wenqin Ai, Dong Liu, Wandong Qi, Mengchen Zhang, Chunyan Yang, Hong Liao

**Affiliations:** ^1^Key Laboratory of Ministry of Education for Genetics, Breeding and Multiple Utilization of Crops, College of Crop Science, Fujian Agriculture and Forestry University Fuzhou, China; ^2^Root Biology Center, Fujian Agriculture and Forestry University Fuzhou, China; ^3^The Key Laboratory of Crop Genetics and Breeding of Hebei, Institute of Cereal and Oil Crops, Hebei Academy of Agricultural and Forestry Sciences Shijiazhuang, China; ^4^Root Biology Center, South China Agricultural University Guangzhou, China

**Keywords:** root architecture, biological nitrogen fixation, soybean, QTLs, synergistic interaction, yield

## Abstract

Soybean [*Glycine max* (L.) Merr] is an important legume crop and its yield largely depends on root architecture (RA) and biological nitrogen fixation (BNF). However, the relationship between RA and BNF, and its genetics behind remain unclear. Here, two soybean genotypes contrasting in RA and their 175 *F*_9:11_ recombinant inbred lines (RILs) were evaluated in field. The shallow-root parent, JD12, had better nodulation and higher yield than the deep-root parent, NF58. Strong correlations between shoot dry weight (SDW) and RA or BNF traits existed in the RILs, and the shallow-root group had more and heavier nodules, as well as higher SDW. After inoculating with rhizobia, roots became shallower and bigger, showing strong synergistic interactions between RA and BNF. In total, 70 QTLs were identified for the 21 tested traits. Among them, *qBNF-RA-C2*, *qBNF-RA-O*, and *qBNF-RA-B1*, were newly identified QTLs for BNF and/or RA traits in soybean, which co-located with the QTLs for SDW detected presently, and with the QTLs for yield identified previously. The results together suggest that there are synergistic interactions between RA and BNF, and the QTLs identified here could be used for breeding new soybean varieties with higher yields through optimization of RA traits and BNF capacity.

## Introduction

Soybean [*Glycine max* (L.) Merr.] is an economically and ecologically important crop that not only provides protein and oil for food and feed, but also serves as a key source of green manure in agro-ecosystems due to its having the highest capacity of biological nitrogen fixation (BNF) observed among leguminous crops (Coale et al., 1985; Kumudini et al., 2008). BNF is a process by which plants, in association with microbes, convert atmospheric nitrogen (N_2_) into ammonia (NH_3_), which is readily available for plant growth. It is estimated that BNF provides 50–70 million tons of N for agricultural systems each year, and, thus, might be the most important source of N for agro-ecosystems (Herridge et al., 2008). In Brazil, over 70% of the N required for soybean growth is derived primarily from BNF (Peoples et al., 2009). Furthermore, a large proportion of the N_2_ fixed by nodules in soybean is available for the growth of subsequent crops in rotation systems, and thereby, makes the soybean-rhizobia symbiosis an efficient way to sustain agricultural development (Qin et al., 2012; Udvardi and Poole, 2013; Pandey et al., 2016). Therefore, breeding elite soybean cultivars that optimize BNF could be an important component of producing high yielding crops while maintaining agriculture sustainability.

Root system is the main organ involved in acquisition of nutrients and water, and is, therefore, a worthwhile subject of research efforts to improve crop yield and adaptation to marginal soils (de Dorlodot et al., 2007; Magalhaes et al., 2007; Tester and Langridge, 2010; Gamuyao et al., 2012; Lynch and Brown, 2012). Due to technical limitations and labor costs, progress in root studies has lagged behind shoot research (Epstein, 2004). Only in recent years, some elite root traits have been introduced in modern breeding programs (Gamuyao et al., 2012; Lynch and Brown, 2012). Among root traits, root architecture (RA), the 3-dimensional spatial configuration of root systems, is particularly critical for root functions in challenging environments (Topp et al., 2013; Li et al., 2016). For example, a deep root system is essential for crops to utilize nitrate and water in deeper soil layers, and is, therefore, beneficial for drought tolerance, particularly under N deficient conditions (Jordan et al., 1983; Wiesler and Horst, 1994). On the other hand, a shallow root system with enhanced adventitious rooting to increase top soil foraging is important for crops to absorb relatively immobile nutrients, such as phosphorus (P) (Lynch, 2011; Li et al., 2016). Developing varieties with RAs suitable for the given field conditions promises to be a sustainable and economical approach to increase crop nutrient efficiency and improve adaptation to stresses.

Nodules and roots are physically closely connected to each other, which leads to strong genetic interactions throughout nodule development and performance of N_2_ fixation and transport functions. Few studies have been performed to simultaneously explore the relationship and the genetics behind BNF and RA traits. It has been reported that soybean roots become shorter after inoculation with rhizobia (Li et al., 2016), possibly due to the substantial demand for carbohydrates and nutrients during nodulation and BNF (Aleman et al., 2010; Reich et al., 2013). *GmEXPB2*, a gene encoding cell wall protein, has been functionally proven to be involved in regulation of both nodulation and RA remodeling in soybean (Guo et al., 2011; Li et al., 2016). Overexpression of *GmEXPB2* not only significantly promoted root growth but also increased nodule number and size, suggesting that the synergistic interactions between RA and BNF might be existed in association with *GmEXPB2* regulation. However, the genetic basis behind the RA and BNF is still uncovered.

In modern breeding programs, identification of QTLs associated with desirable traits is being increasingly utilized in marker-assisted selection (MAS) and gene discovery (Kumar et al., 2017; Lu et al., 2017). There are many successful examples of using QTLs to increase quality, yield and disease resistance in cereal crops, such as rice (Takagi et al., 2013; Ali et al., 2017), maize (Mesterházy et al., 2012; Wu et al., 2016) and wheat (Tian et al., 2007; Zhang et al., 2017), as well as, in legumes (Wu et al., 2016; Lu et al., 2017). Many root traits are complex and expected to be controlled by many loci/genes, exhibiting highly flexible responses to environmental conditions (López-Bucio et al., 2003). In recent years, many quantitative trait locus (QTLs) for RA traits have been mapped in cereal crops, such as in rice (Ding et al., 2011; Topp et al., 2013), wheat (Ren et al., 2012; Christopher et al., 2013) and maize (Ruta et al., 2010; Burton et al., 2014). However, information on QTLs for RA traits remains scarce for soybean, with only two such published reports incorporating field evaluation (Liang et al., 2010; Abdel-Haleem et al., 2011). QTLs for BNF associated traits have been identified in several legumes, including common bean (Tsai et al., 1998; Souza et al., 2000), pea (Bourion et al., 2010) and *Lotus japonicus* (Tominaga et al., 2012). Even BNF related traits have been genetically mapped in soybean, albeit in greenhouse experiments with limited genome coverage (Tanya et al., 2005; Nicolás et al., 2006; Santos et al., 2013), and no QTLs for BNF traits have yet been successfully applied in soybean breeding programs for improving BNF capacity and yield.

In order to better incorporate BNF and RA traits into breeding programs aiming to facilitate nutrient uptake from soils and N_2_ fixation from atmosphere, as well as to improve soybean yields, researchers need to increase understanding of relationship between RA and BNF, and its genetics behind individual traits. In the present study, two soybean genotypes contrasting in RA and their 175 *F*_9:11_ recombinant inbred lines (RILs) were grown in the field to evaluate the relationships among BNF, RA and plant growth, as well as to identify QTLs for associated traits under conditions of rhizobial inoculation and non-rhizobial inoculation.

## Materials and Methods

### Plant Materials and Field Trails

Two soybean [*G. max* (L.) Merr] cultivars, JD12 and NF58, were crossed to develop RILs using single seed descent (SSD). In total, 175 *F*_9:11_ RILs were derived to construct a genetic linkage map and detect QTLs associated with BNF and RA traits, as well as shoot dry weight (SDW). Two field trials were conducted in the Dishang experimental farm (E114.48°, N38.03°) of the Institute of Cereal and Oil Crops, Hebei Academy of Agricultural and Forestry Sciences, Shijiazhuang City, Hebei Province, China. The soil in the experiment site belongs to Fluventic Ustochrept soils. Basic characteristics of the top 30-cm soils in field were as follows: pH, 8.5; organic matter, 20.6 g.kg^-1^; available P (Olsen-P), 14.5 mg. kg^-1^; available N, 81.2 mg. kg^-1^; and available K, 159.3 mg kg^-1^. The previous crop was wheat with 900 kg/ha of compound fertilizer (N:P_2_O_5_:K_2_O = 15:15:15) as base fertilizer and 450 kg/ha of urea as additional fertilizer during elongation stage. No fertilizer was applied during soybean growth. One trial was managed with rhizobial inoculation (+R) as described by Qin et al. (2012), and another one was run under natural conditions without inoculation (-R). The soybean plants were irrigated three times, including before planting, at flowering and seed filling stage following the local practice.

Parental genotypes and RILs were planted in a split plot design with plots arranged in randomized complete blocks within each block of split plots. There were nine replications for parental genotypes and three replications for RILs, and in total, were 1086 plots in the field. Thirty seeds were sown per plot in three 1.5 m rows spaced 0.5 m apart.

### Plant Sampling and Measurements

At R6 stage, three representative plants from each plot, in total 3258 plants, were extracted manually from the soil with root systems largely intact. Entire root systems were dug out and carefully cleaned as described by Liang et al. (2010), and all nodules were removed prior to digitally scanning root systems. The root systems of the nine plants for each genotype were visually rated according to Zhao et al. (2004) on scale of 1–3; where 1 is a shallow RA when the basal root growth angle of most basal roots was less than 40 degrees from horizontal, and 3 is a deep RA when more than 60 degrees from horizontal, and 2 is an intermediate RA (type 2) between type 1 and 3. Digital images were quantified with computer image analysis software (Win-RhizoPro, Régent Instruments, Ville de Québec, QC, Canada) for RA traits. Roots were separated into three groups based on the root diameter (RD), including fine (RD < 1 mm), medium roots (1 mm ≤ RD < 2 mm) and coarse roots (RD ≥ 2mm). Root length (RL), root surface area (RSA), root volume (RV) were quantified from each group. Since soybean nodule growth is determinate, nodule size is correlated with nodule age. Large (diameter ≥ 2 mm) and small (diameter < 2mm) nodules were separated using a mesh. In total, 21 traits were investigated, including 6 BNF traits: number (NTN, NBN, and NSN) and dry weight (WTN, WBN, and WSN) of total, big and small nodules; 14 RA traits: average root diameter (ARD) and total root dry weight (RDW); and RL, RSA, RV for total (TRL, TRSA, and TRV), fine (FRL, FRSA, and FRV), medium (MRL, MRSA, and MRV) and coarse roots (CRL, CRSA, and CRV); and SDW.

### Genetic Linkage Map Construction

The parental genotypes, JD12 and NF58, were first surveyed for 347 simple sequence repeat (SSR) markers selected from an integrated soybean genetic linkage map (Cregan et al., 1999; Song et al., 2004). Primer sequences were obtained from the SoyBase website^1^. DNA was extracted from fresh leaf tissues of 175 *F*_9:11_ RILs at V3 stage according to SDS methods as described previously (Hnetkovsky et al., 1996) with slight modifications. All DNA samples were diluted using 200 μl 1 × TE buffer (pH 8.0) prior to PCR amplification. Polymerase chain reaction conditions were as follows: denaturation at 92°C for 10 min; 35 cycles of denaturation at 92°C for 30 s, annealing at 52°C for 30 s, extension at 72°C for 30 s, and a final extension at 72°C for 10 min before cooling to 4°C. PCR products were visualized after electrophoresis on an 8% polyacrylamide gel followed by silver staining. Products with sizes consistent with those from either JD12 or NF58 were recorded as A or B. An expected ratio of 1:1 was used to detect the segregation of products in the *F*_9:11_ RIL population. Chi-square (χ^2^) analysis was performed to test goodness-of-fit of the observed vs. expected segregation ratios. When observations produced one degree of freedom, then the Yates correction factor (Yates, 1934) was applied in the Chi-square (χ^2^) calculation.

The QTL IciMapping V4.1 software was used for linkage map construction (Meng et al., 2015). In general, there are three steps, grouping, ordering and rippling. Marker grouping was based on: (i) logarithm-of-odds (LOD) scores exceeding 3, (ii) a recombination frequency > 0.3, (iii) a marker distance < 50, (iv) a predefined group number, and (v) anchored marker information. The order of markers was first calculated using nnTwoOpt algorithms (Li et al., 2008) and checked by SER (Buetow and Chakravarti, 1987) and RCORD (Van et al., 2005) methods.

### QTL Detection

QTL detection was performed by using QTL IciMapping V4.1 software (Meng et al., 2015) at 1.0 cM intervals to map QTLs to the SSR map of the RIL population. QTLs were detected successively by inclusive composite interval mapping (ICIM) (Meng et al., 2015). Each LOD score larger than 2.5 was considered as resulting from the presence of a QTL. The QTL additive effects (Add) were also estimated by ICIM methods, where an additive effect represents the mean effect of the replacement of the JD12 allele by the NF58 allele at a particular locus. An Add-value > 0 or <0 indicates effects derived from JD12 or NF58, respectively. Phenotypic variation explained (PVE) by a single QTL was calculated as follows: PVE (%) = Vg/Vp^∗^100%, Vg = Add^∗^Add, Vp stands for total variation.

### QTL Comparison

In order to compare the location of the QTLs identified in this study with related QTLs reported in previous studies, very closely linked, same located and containing overlap region QTLs were first integrated and then integrated QTLs were projected onto the soybean consensus map at SoyBase^1^ based on flanking marker information in common between this study used and the consensus map. The graphical presentation of linkage maps and QTLs were drawn using MapChart 2.2 software (Voorrips, 2002).

### Statistical Analysis

Data for all 21 measured traits were used for variance, correlation and QTL analyses. Analysis of variance (ANOVA) were implemented in the QTL IciMapping V4.1 software (Meng et al., 2015). Parental genotypes were also planted with nine repetitions in both trials, which were analyzed separately from the RILs. Broad sense heritability (*h*^2^_b_) was estimated for each trait according to: *h*^2^_b_ = VG/(VG + VE), where VG was the variance between RILs, and VE was the variance within RILs. The student’s *t*-test was used to test for significance effects of rhizobial inoculation on each trait in RILs.

## Results

### Root Architecture (RA), Biological Nitrogen Fixation (BNF) and Growth of Two Parental Genotypes

**FIGURE 1:**
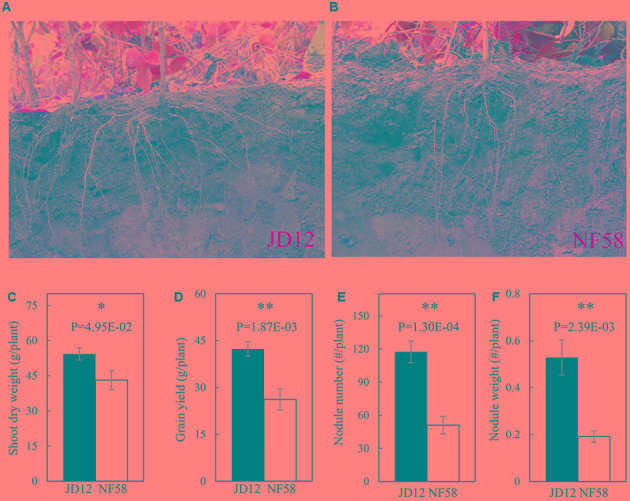
Root architecture, biological nitrogen fixation (BNF) and growth of JD12 and NF58 in the field. **(A)** Photo of JD12 roots [shallow root architecture); **(B)** photo of NF58 roots (deep root architecture (RA)]; **(C)** shoot dry weight (SDW); **(D)** grain yield; **(E)** nodule number; **(F)** nodule weight. Bars represent means ± SE from nine replications. Asterisks indicate the significance of differences between JD12 and NF58 through Student *t*-test at 5% (^∗^) and 1% (^∗∗^) level.

The two parental genotypes, JD12 and NF58, showed contrasting RA and growth performance in the field (**Figure 1**). The growth angles of most basal roots of JD12 were lower than 40°, and thus JD12 was considered having a shallow RA and its RA value was recorded as 1. On the other hand, the angles of most basal roots of NF58 were more than 60°, and therefore, NF58 had a deep RA and its RA value was 3. Furthermore, JD12 had a bigger shoot and higher yield than NF58 as indicated by 25.82 and 61.60% more SDW and grain yield than NF58 (**Figures 1C,D**), respectively. However, the two parental genotypes did not significantly differ in total root length (TRL) and RDW (Supplementary Figure S1), suggesting that the better growth of JD12 might be attributed to its shallow RA. Interestedly, the shallow-root parent JD12 also had more and heavier nodules than the deep-root parental genotype, NF58 (**Figures 1E,F**), implying that there might be synergistic interactions between RA and BNF.

### Effects of Root Architecture and Rhizobial Inoculation on the Growth of RILs

**FIGURE 2:**
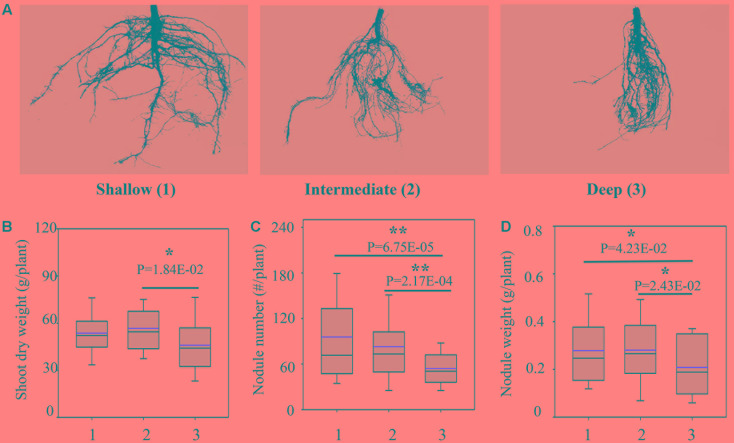
Biological nitrogen fixation traits as affected by RA in the RILs. **(A)** Representative pictures of root systems from different groups; 1, 2, and 3 represent the group of RILs with shallow, intermediate and deep RA, respectively; **(B)** SDW; **(C)** nodule number; **(D)** nodule weight. The black and red lines, lower and upper edges, and bars up or low side the boxes represent median and mean values, 25th and 75th, 5th and 95th percentiles of all data, respectively. Asterisks indicate the significance of differences between shallow, intermediate and deep group through Student *t*-test at 5% (^∗^) and 1% (^∗∗^) level.

Since the RA was significantly segregated in the RILs, we classified the RILs into three groups, including shallow, intermediate and deep groups, representing the progenies having shallow RA, intermediate RA and deep RA as defined above (**Figure 2A**). Compared with the deep group, the shallow and intermediate groups exhibited greater yield potentials and higher BNF capacities, as indicated by the combination of higher SDW, more and heavier nodules (**Figures 2B–D**). However, the three groups did not significantly differ in TRL and RDW (Supplementary Figure S2), further suggesting that the better growth and BNF capacity of RILs might be attributed to the shallow RA.

**FIGURE 3:**
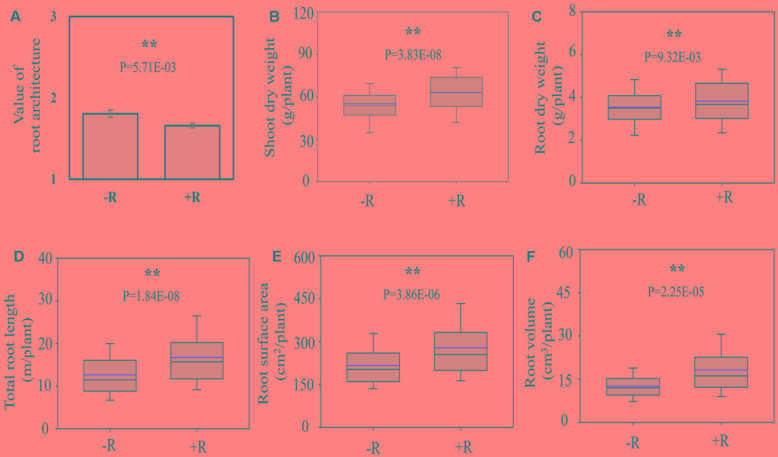
Root architecture traits as affected by rhizobial inoculation in175 *F*_9:11_ soybean recombinant inbred lines (RILs) under field conditions. **(A)** Value of RA; **(B)** shoot dry weight (SDW); **(C)** root dry weight (RDW); **(D)** total root length (TRL); **(E)** root surface area (RSA); **(F)** root volume (RV). Value of shallow, intermediate and deep RA was recorded as 1, 2, and 3 (see Materials and Method for details), respectively. “–R” and “+R” represent without and with rhizobial inoculation, respectively. The black and red lines, lower and upper edges, and bars up or low side the boxes represent median and mean values, 25th and 75th, 5th and 95th percentiles of all data, respectively. Asterisks indicate the significance of differences between –R and +R conditions through student *t*-test at 5% (^∗^) and 1% (^∗∗^) level, respectively.

Inoculation with rhizobia significantly affected the RA and plant growth. After inoculating with effective rhizobial strains, the roots of RILs became shallower and bigger, as supported by significantly lower average value of RA of RILs, and higher RDW, longer TRL as well as more RSA and RV (**Figure 3**). Meanwhile, the average SDW of RILs was also increased 18.25% by rhizobial inoculation, showing that the synergistic interactions existed between RA and rhizobial inoculation, and which might positively contribute to soybean plant growth.

### Phenotypic Variations among Recombinant Inbred Lines

In the field trails, the phenotypic variation was evaluated for 21 traits, including 6 associated with BNF, 14 with RA and SDW. Means and range for the 21 traits are summarized in **Table 1** under natural conditions. Within the 175 *F*_9:11_ soybean RILs, significant phenotypic variations existed for all the 21 traits. The mean value for each trait among all RILs was between the mean values of the two parents, while the maximum and minimum values were beyond the extremes of the two parents. Except for the two BNF traits defined for small nodules (NSN and WSN), distributions for the other 19 traits were approximately normal according to Kurtosis and Skewness values calculated over three replicates. Broad-sense heritability (*h*^2^_b_) for the 21 traits varied from 0.59 to 0.89, with higher values generally observed for BNF traits than RA traits and SDW (**Table 1**). This was also supported by the phenotypic variation and genetic analysis of the same traits under the conditions with rhizobial inoculation (Supplementary Table S1). After inoculating with rhizobia, the means, Kurtosis and Skewness values and *h*^2^_b_ for 6 BNF traits did not change significantly, while the 14 RA traits and SDW were significantly promoted, providing additional evidence that BNF had higher heritability than RA and plant growth.

**Table 1:** Phenotypic variation and genetic analysis of 21 traits using 175 *F*_9:11_ soybean recombinant inbred lines (RILs) under natural conditions in the field.

Traits	Parents		RILs							
	JD12	NF58	Maximum	Minimum	Mean	*SD*	CV/%	Kurt	Skew	*h*^2^_b_
NTN	117.50	51.00	331.67	4.38	83.40	55.77	66.87	4.29	1.79	0.89
WTN	0.53	0.19	0.81	0.01	0.27	0.15	56.91	0.34	0.70	0.84
NBN	85.25	35.89	162.22	1.00	48.56	29.89	61.56	1.29	1.12	0.86
WBN	0.50	0.18	0.66	0.01	0.23	0.13	57.70	-0.18	0.54	0.82
NSN	32.25	15.11	232.00	2.57	34.84	32.54	93.40	9.84	2.66	0.89
WSN	0.03	0.01	0.28	0.00	0.04	0.04	94.43	13.39	3.02	0.87
RDW	3.68	3.20	5.63	1.37	3.54	0.93	26.39	-0.53	0.04	0.85
SDW	59.16	45.26	89.59	23.07	53.76	12.71	23.64	-0.03	-0.09	0.72
TRL	1086.20	1958.65	3207.23	227.40	1261.36	530.13	42.03	1.02	0.96	0.68
FRL	959.99	1740.02	2984.07	210.39	1129.42	499.47	44.22	1.15	1.01	0.67
MRL	100.91	154.12	186.47	8.99	98.30	35.68	36.30	-0.55	0.20	0.70
CRL	24.33	62.28	76.47	7.76	31.42	12.10	38.52	0.98	0.87	0.59
TRSA	198.01	369.20	420.97	34.95	216.75	71.96	33.20	-0.07	0.57	0.66
FRSA	91.49	182.05	224.98	18.88	105.36	40.39	38.33	0.60	0.86	0.65
MRSA	42.57	65.80	79.16	3.87	41.82	15.50	37.05	-0.54	0.22	0.70
CRSA	37.99	77.75	131.71	8.24	47.98	16.54	34.47	3.24	1.07	0.63
TRV	10.05	16.50	40.32	1.08	12.62	4.72	37.44	5.91	1.35	0.68
FRV	1.07	2.18	2.65	0.18	1.22	0.44	35.84	0.55	0.79	0.65
MRV	1.48	2.32	2.77	0.14	1.47	0.56	37.89	-0.52	0.24	0.70
CRV	7.50	11.99	35.99	0.76	9.93	4.13	41.58	8.15	1.70	0.64
ARD	0.68	0.62	0.91	0.37	0.59	0.12	19.91	-0.25	0.45	0.70

*RILs: recombinant inbred lines; roots were separated into three groups based on the root diameter (RD), including fine (RD < 1 mm), medium roots (1 mm ≤ RD < 2 mm) and coarse roots (RD ≥ 2 mm). Twenty-one traits included six BNF (biological nitrogen fixation) traits: NTN (number of total nodule, #/plant), WTN (weight of total nodule, g/plant), NBN (number of big nodules, #/plant), WBN (dry weight of big nodules, g/plant), NSN (number of small nodules, #/plant) and WSN (dry weight of small nodules, g/plant); 14 RA (root architecture) traits: TRL (total root length, cm/plant), FRL (fine root length, cm/plant), MRL (medium root length, cm/plant), CRL (coarse root length, cm/plant), TRSA (total root surface area, cm^2^/plant), FRSA (surface area of fine roots, cm^2^/plant), MRSA (surface area of medium roots, cm^2^/plant), CRSA (surface area of coarse roots, cm^2^/plant), TRV (total root volume, cm^3^/plant), FRV (fine root volume, cm^3^/plant), MRV (medium root volume, cm^3^/plant), CRV (coarse root volume, cm^3^/plant), ARD (average root diameter, mm) and RDW (root dry weight, g/plant); and SDW (shoot dry weight g/plant).*

Except for ARD, all traits were significantly correlated with each other (Supplementary Table S2). More importantly, all BNF and RA traits, were significantly positively correlated with SDW under both rhizobial inoculation conditions. This also suggested that BNF and RA might positively contribute to plant growth. Furthermore, the successively high correlations of RDW, WBN, WTN, and NBN with SDW suggested that these were highly important among all of the tested BNF and RA traits for plant growth. Finally, although the correlation coefficients among the 21 traits were somewhat affected by rhizobial inoculation, the overall importance of BNF remained consistent even without inoculation.

### Genetic Linkage Map Construction

An expected, most of the observed markers segregated in 1:1 ratios in the RIL population. Results from χ^2^ testing indicated that 109 of 133 (82.0%) SSR markers segregate in 1:1 ratios and were revealed as co-dominant markers. After construction of the genetic map, unlinked markers were manually adjusted based on marker information (Cregan et al., 1999; Song et al., 2004). 133 SSR markers were finally grouped into 20 linkage groups with a total length of 2069.77 cM (Supplementary Figure S3), which collectively covered more than 60% of the consensus soybean linkage map at SoyBase^2^. Each group was covered by SSR markers from 28.69 to 160.48 cM, and the average distance between the markers was 15.56 cM. The order and distance of most of the SSR markers in our constructed map were consistent with the soybean consensus genetic linkage map from SoyBase^2^.

The *T* gene located on chromosome 6 (LG:C2) has been identified as responsible for pubescence color (Yang et al., 2002). In order to examine the fidelity of the genetic map constructed herein, this pubescence color gene was mapped relative to the markers used in the current study. As expected, the gene encoding pubescence color was mapped as the *T* gene located on chromosome 6 between marker satt286 and satt557 with a high LOD value of 21.74 (Supplementary Figure S3). Taken together, the above results clearly indicated that the linkage map constructed in this study had high quality and could be used in further studies.

### QTL Identification

A total of 70 significant QTLs were identified for 20 of the 21 tested traits (**Tables 2**, **3**), and explained 3.4–25.7% of the phenotypic variation observed among the 175 *F*_9:11_ soybean RILs grown in the field. Among them, 43 QTLs were for RA traits (**Table 2**), and 24 and 3 for BNF and SDW (**Table 3**), respectively. Most of the QTLs could be grouped into three loci, and thereby named as *qBNF-RA-C2*, *qBNF-RA-O*, and *qBNF-RA-B1*, respectively (Supplementary Figure S4). These three loci could explain almost all phenotypic variations for the 20 tested traits. For example, *qBNF-RA-C2* on the chromosome 6, explained the genetic variations for 31 traits, with the percentage of variation explained ranging from 7.68% (-NBN) to 25.70% (+RDW), and with LOD values ranging from 4.57 (+FRV) to 18.88 (+RDW). *qBNF-RA-O* on the chromosome 10, accounted for phenotypic variations of 24 traits, with percentages explained ranging from 3.43% (-WBN) to 12.86% (+NBN), and with LOD values between 2.71 (-SDW) and 9.67 (+RDW). *qBNF-RA-B1* on the chromosome 11, mainly explained variations in observed BNF traits, with percentages explained ranging from 6.80% (+NTN) to 11.32% (-WBN), but, it also accounted for 5.25% of the variation in +RDW. Moreover, integrated QTLs on LG:N (*qRA-N*) could explain five RA traits ranging from 4.90% (-TRSA) to 8.24% (-TRL) under without rhizobial inoculation field conditions. Additionally, both *qRA-L* and *qRA-I* were QTLs for RDW and cloud explain 6.35 and 6.79% variations under with and without rhizobial inoculation conditions, respectively (**Table 2**).

**Table 2:** Putative QTLs detected for RA traits using 175 *F*_9:11_ soybean RILs in the field.

Interval	Integrated QTL	Separate QTL	Chr	Position	LOD	PVE (%)	Add
Satt660-Satt312	*qRA-N*	*q*-*FRL*	3	72	3.15	8.11	-147.86
		*q*-*FRSA*	3	72	3.10	7.87	-12.00
		*q*-*FRV*	3	72	2.82	7.17	-0.12
		*q*-*TRL*	3	72	3.22	8.24	-158.69
		*q*-*TRSA*	3	72	2.81	4.90	-18.71
Satt286-Satt289	*qRA-C2*	*q*-*MRL*	6	72	5.44	9.79	13.28
		*q*-*MRSA*	6	72	5.61	10.07	5.83
		*q*-*TRSA*	6	72	5.15	8.97	25.37
		*q*-*MRV*	6	73	5.77	10.09	0.21
		*q*-*TRV*	6	74	7.05	14.61	1.91
		*q*-*CRSA*	6	75	5.00	12.56	5.83
		*q*-*CRV*	6	75	4.86	12.19	1.43
		*q+CRL*	6	76	9.97	18.13	10.64
		*q+CRSA*	6	76	9.66	17.88	13.55
		*q+CRV*	6	76	8.01	15.37	2.94
		*q+FRL*	6	76	6.23	14.98	243.26
		*q+FRSA*	6	76	5.28	12.88	20.16
		*q+FRV*	6	76	4.57	11.25	0.21
		*q+MRL*	6	76	7.17	17.04	22.20
		*q+MRSA*	6	76	8.92	17.85	10.59
		*q+MRV*	6	76	9.17	18.61	0.39
		*q+RDW*	6	76	18.88	25.70	0.63
		*q+TRL*	6	76	6.60	15.80	276.01
		*q+TRSA*	6	76	7.01	16.69	45.54
		*q+TRV*	6	76	8.69	16.30	3.55
		*q*-*CRL*	6	76	4.87	12.17	4.24
		*q*-*RDW*	6	76	8.90	14.74	0.39
Satt477-Sat_190	*qRA-O*	*q*-*MRL*	10	124	4.23	10.07	-13.49
		*q*-*MRSA*	10	124	4.28	10.24	-5.89
		*q*-*MRV*	10	124	4.33	10.61	-0.21
		*q*-*TRSA*	10	126	4.59	11.79	-29.14
		*q+TRV*	10	138	3.42	6.93	-2.33
		*q+CRV*	10	139	3.59	6.92	-1.99
		*q*-*RDW*	10	143	7.14	12.06	-0.36
		*q+MRV*	10	144	2.63	4.91	-0.20
		*q+RDW*	10	144	9.67	11.75	-0.43
		*q*-*TRV*	10	144	2.80	5.54	-1.19
		*q+MRSA*	10	145	2.53	5.10	-5.71
		*q+CRL*	10	146	4.04	8.38	-7.29
		*q+CRSA*	10	146	3.66	7.56	-8.87
Satt509-Sat_272	*qRA-B1*	*q+RDW*	11	31	3.76	5.25	0.28
Sat_286-satt229	*qRA-L*	*q+RDW*	19	110	4.33	6.35	-0.32
Satt571-Satt496	*qRA-I*	*q*-*RDW*	20	14	3.30	6.79	0.27

*RA: root architecture; N, C2, O, B1, L, and I represented the name of the linkage group where the integrated QTLs were located, respectively. “-” and “+” means the QTL was identified under without and with rhizobial inoculation conditions, respectively. Add value > 0 and < 0 stand for increasing effects of the QTLs derived from JD12 and NF58, respectively.*

**Table 3:** Putative QTLs detected for biological nitrogen fixation (BNF) traits and SDW using 175 *F*_9:11_ soybean RILs in field.

Interval	Integrated QTL	Separate QTL	Chr	Position	LOD	PVE(%)	Add
Satt286-Satt281	*qBNF-C2*	*q*-*NBN*	6	78	5.22	7.68	11.28
		*q+NBN*	6	76	8.34	10.11	14.22
		*q*-*WBN*	6	80	5.12	9.62	0.05
		*q+WBN*	6	79	8.35	13.56	0.08
		*q*-*SDW*	6	73	8.48	16.06	5.96
		*q+SDW*	6	76	6.37	15.29	7.50
		*q+NTN*	6	76	7.58	11.12	23.54
		*q*-*WTN*	6	79	5.16	9.20	0.06
		*q+WTN*	6	79	8.77	13.92	0.09
Satt592-Sat_190	*qBNF-O*	*q*-*NBN*	10	150	5.09	8.66	-12.09
		*q+NBN*	10	148	7.89	12.86	-16.14
		*q*-*WBN*	10	142	2.74	3.40	-0.03
		*q+WBN*	10	143	4.32	5.38	-0.05
		*q+NSN*	10	148	3.10	6.13	-10.31
		*q+WSN*	10	149	2.75	5.77	-0.01
		*q*-*SDW*	10	141	2.71	4.26	-3.09
		*q*-*NTN*	10	150	2.68	5.67	-18.41
		*q+NTN*	10	143	7.34	11.94	-24.59
		*q*-*WTN*	10	142	3.47	4.54	-0.04
		*q+WTN*	10	143	5.24	6.47	-0.06
Satt509-Sat_272	*qBNF-B1*	*q*-*NBN*	11	28	5.24	8.55	11.91
		*q+NBN*	11	28	6.41	10.28	14.33
		*q*-*WBN*	11	28	6.35	11.32	0.06
		*q+WBN*	11	26	5.88	10.76	0.08
		*q+NTN*	11	31	4.15	6.80	18.43
		*q*-*WTN*	11	29	6.20	11.05	0.07
		*q+WTN*	11	26	6.20	11.21	0.08

*BNF: biological N_2_ fixation; N, C2, O, B1, L, and I represented the name of the linkage group where the integrated QTLs were located, respectively “-” and “+” means the QTL was identified under without and with rhizobial inoculation conditions, respectively. Add value > 0 and < 0 stand for increasing effects of the QTLs derived from JD12 and NF58, respectively.*

### Co-location with Previously Reported Yield, BNF and RA Traits

**FIGURE 4:**
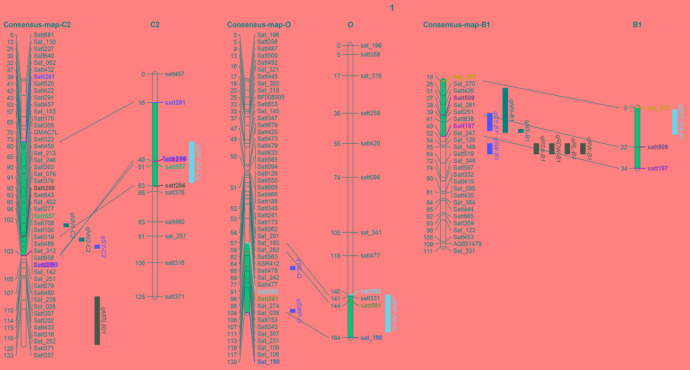
Co-location analysis using three QTL clusters (pink) detected here, along with previously identified QTLs for yield (red), BNF (black) and RA (green) traits. Previously reported QTLs and references are showed as follows: *qSY-C2* (Kabelka et al., 2004), *qYIE- O* (Wang et al., 2014), *qSWP-O* (Liu et al., 2011), *qSW-B1* (Liang et al., 2010) and *qSY-B1* (Du et al., 2009) for yield and SDW; *qNN1-C2* (Hwang et al., 2014), *qNN2-C2* (Hwang et al., 2014), *qNN-B1* (Santos et al., 2013) and *qNS-B1* (Hwang et al., 2014) for BNF traits; *qRSA-B1*, *qRDW-B1*, *qRL-B1*, and *qRW-B1* for RA traits (Liang et al., 2010). Projected regions are highlighted in dark blue. Markers in different colors indicate the corresponding markers on LGs and in projection regions.

One of the most important breeding goals for all crops is to achieve high yields. In order to analyze relationships between the QTLs identified in this study to QTLs previously detected for yield and BNF, as well as RA, the QTLs identified in the present study were projected onto the soybean consensus map^2^. Three QTL clusters, *qBNF-RA-C2*, *qBNF-RA-O*, and *qBNF-RA-B1*, were found to co-localize with several previously identified QTLs for yield, BNF and RA traits (**Figure 4**). This included that *qBNF-RA-C2*, *qBNF-RA-O*, and *qBNF-RA-B1* co-localized with the reported yield QTLs *qSY-C2* (Kabelka et al., 2004), *qYIE-O* (Wang et al., 2014) *qSWP-O* (Liu et al., 2011), and *qSY-B1* (Du et al., 2009), while *qBNF-RA-C2* and *qBNF-RA-B1* co-localized with the previously identified BNF QTLs *qNN1-C2*, *qNN2-C2*, and *qNN-B1* for nodule number (Santos et al., 2013; Hwang et al., 2014), along with *qNS-B1* for nodule size (Hwang et al., 2014). While no previous RA QTLs co-localized with the currently identified RA QTLs, four RA QTLs (*qRSA-B1*, *qRDW-B1*, *qRL-B1*, and *qRW-B1*) and 1 yield QTL (*qSW–B1*) identified by Liang et al. (2010) were located relatively close to the presently identified QTL cluster *qBNF-RA-B1*. On the other hand, the previously identified locus on LG I for BNF traits (Hwang et al., 2014) was not identified in the current population. Instead, a new QTL cluster was identified (*qBNF-RA-O*) on LG O for –NBN, +NBN, -WBN, +WBN, +NSN, and +WSN, which also co-located with SDW in this study, as well as, a yield QTL from previous studies as described above (**Figures 3**, **4**). The co-location of the QTLs for BNF and/or RA traits with SDW detected in this study, and with the previously identified QTLs for soybean yield, strongly supported that the genetic links of BNF and RA traits with growth and yield existed in soybean; and the QILs identified in this study could be of great interest for breeders to develop new soybean varieties with higher yields through optimization of RA traits and BNF capacity.

## Discussion

Increasing demand for food driven by growing populations and rising requirements for better living environments place demands on agriculture to increase productivity while also being sustainable. Legumes play critical roles in ensuring global food security and improving soil quality through BNF, the unique ability to fix atmospheric nitrogen (N_2_) into ammonia by rhizobia in the special symbiotic organ, nodules (Udvardi and Poole, 2013). Therefore, developing cultivars with higher yield potentials and superior BNF capacities are main targets in modern legume breeding programs.

The importance of legume breeding in increasing BNF has been highlighted for several decades, yet understanding of the genetics underlying BNF and variations of associated traits under different environmental conditions remains limited (Kouchi et al., 2010; Pandey et al., 2016). Here, an effort to study the relationship between BNF and plant growth, and to identify QTLs associated with BNF traits under two different environmental conditions is presented. All the coefficients of variations for the six BNF traits are larger than 50%, and the *h*^2^_b_ is above 0.8, corresponding to great heritability and variability as previously reported (Souza et al., 2000; Santos et al., 2013). The two regions (LGs B1 and C2) controlling BNF traits identified in this study (**Table 3**), have been previously reported (Tanya et al., 2005; Nicolás et al., 2006; Santos et al., 2013), confirming the reliable association of these regions with BNF traits. Since soybean nodules are of the determinate type, nodule size is closely related to nodule development stage and BNF capacity (Popp and Ott, 2011). Therefore, in order to more precisely analyze BNF capacity, nodules were separated into two groups (big and small) in the current study (**Table 3**). As a result, one new QTL cluster for BNF traits under two field conditions was mapped to LG O. On the other hand, QTLs previously mapped to D1b, H, E and B2 (Nicolás et al., 2006; Santos et al., 2013), on A1, D1b, J and I (Tanya et al., 2005) were not identified in this study. Possible explanations for these discrepancies include the separation of nodules by size herein, or the fact that the LOD threshold used in this study, 2.5, was higher than previously applied thresholds.

Roots are difficult to quantify and study, especially under natural soil conditions. Therefore, root research typically lags behind investigations focusing on shoots (Epstein, 2004). Few root QTL studies have been reported for soybean, particularly under field conditions. In this study, 43 QTLs for RA traits have been identified. Among them, the QTL cluster on LG B1 (**Table 2** and Supplementary Figure S4) is close to two QTL clusters associated with traits for roots and P efficiency detected by Liang et al. (2010). Differences in identified positions of QTLs among research efforts might be attributable to variations in experimental conditions or progress in the coverage of the soybean genome in the time between two studies. Five QTLs for soybean fibrous roots have been identified in the field (Abdel-Haleem et al., 2011), but none of them co-locate with the QTLs for RA in the current study, which could be due to differences in the quantification methods employed to evaluate fibrous roots between this study and that one. A major QTL locus on LG: A2 for root traits, five epistatic QTLs for RDW on LG: D1a, C2, A2, O and H, and 20 QTLs for root traits distributed on 11 LGs have been reported (Liang et al., 2014; Manavalan et al., 2015). However, none of them could be confirmed or associated with nearby RA QTLs in this study, possibly due to differences among growth stages sampled and experimental conditions. It has been reported that RA traits could be dramatically influenced by growth stage and environmental factors, such as aerenchyma formation was a result of flooding (Chimungu et al., 2015), which may affect soil exploration by plant roots (Shimamura et al., 2010) and further indirectly influence QTL identification for RA and BNF traits. For example, nine RA QTLs in soybean have been identified under field conditions (Brensha et al., 2012), but with plants that had been grown in pots for 3 weeks before being transferred into field plots. None of these QTLs is consistent with any QTL identified here. Overall, the above examples demonstrate that RA traits are dramatically influenced by growth stage and environmental conditions, especially soil physical and chemical conditions, along with the biological communities harbored in these soils (Hovey, 2012; Brown et al., 2013; Saito et al., 2014).

Given the intimate contact between roots and soils, it is not surprising that roots are sensitive to variations in soil conditions and microbial interactions (Wang et al., 2011; Brown et al., 2013; Hossain, 2015). Rhizobia are able to interact symbiotically with legume roots, which is, therefore, likely to significantly impact RA traits in this group of plants. Even so, there is a notable lack in reports on the effects of rhizobial inoculation on RA traits. In this study, soybean plants were grown in the field with (+) and without (-) rhizobial inoculation. As expected, all of the 14 observed RA traits were significantly altered by rhizobial inoculation (Supplementary Table S1 and **Figure 3**), which resulted in bigger root system with the *P*-values of all the tested RA traits between two treatments reached to significant level. Even though RA traits were typically controlled by the same QTL regardless of rhizobial inoculation (**Tables 2**, **3** and Supplementary Figure S4), including, for example the mapping of RL and RDW QTLs to LGs C2 and O, a number of unique QTLs were found only for specific combinations of RA traits and rhizobial inoculation, such as *qRA-L* or *qRA-I* for RDW with or without rhizobial inoculation; and *qRA-N* for TRL, TRSA, FRL, FRSA, and FRV under natural conditions without rhizobial inoculation (**Table 2**). However, the LOD values of these RA QTLs were relatively low, which indicates strong environmental effects that need to be further evaluated.

The only QTL study published on both BNF and RA traits in legumes has identified a significant positive relationship between nodule establishment and pea root system growth through QTL analysis (Bourion et al., 2010). Co-localization of the QTLs for pea BNF and RA traits suggests that these loci are tightly linked and each contributes to N acquisition efficiency, N and C accumulation, and plant development (Bourion et al., 2010). In the current work with soybean, most BNF traits acted in close conjunctions with RA traits, and some of them co-located to the same QTLs, including *qBNF-RA-C2*, *qBNF-RA-O*, and *qBNF-RA-B1* (**Tables 2**, **3** and Supplementary Figure S4). This strongly suggests that BNF and RA traits are genetically linked, and its underlying mechanisms deserve further studies at both physiological and molecular levels. It is worthwhile to particularly emphasize that two important QTL clusters identified herein (*qBNF-RA-C2* and q*BNF-RA-O*) not only co-localize with the QTLs for SDW identified in this study, but also with previously detected QTLs for soybean yield (Kabelka et al., 2004; Liu et al., 2011; Wang et al., 2014). Plus, *qBNF-RA-B1* co-localizes with yield QTL identified by Du et al. (2009). The co-location of QTLs for yield, BNF and RA traits strongly suggests that BNF and RA related traits can affect soybean yield and might be considered as important breeding targets in programs seeking to develop elite genotypes producing higher yields through optimization of RA traits and BNF capacity.

## Author Contributions

YY and HL designed the experiments and analyzed the data. YY, QZ, XL, WA, DL, and WQ carried out the experiments. CY, MZ, and QZ constructed and genotyped the RILs. HL and YY wrote the paper.

## Conflict of Interest Statement

The authors declare that the research was conducted in the absence of any commercial or financial relationships that could be construed as a potential conflict of interest. The reviewer KL and handling Editor declared their shared affiliation, and the handling Editor states that the process met the standards of a fair and objective review.

## Footnotes

1 https://www.soybase.org/

2 https://soybase.org
