# Supplementary material for: Characterization of Genetic Basis on Synergistic Interactions between Root Architecture and Biological Nitrogen Fixation in Soybean
Source: Front Plant Sci. 2017 Aug 23;8:1466. doi: 10.3389/fpls.2017.01466 (PMC5572596; doi:10.3389/fpls.2017.01466)
Supplement: Supplementary file 1 [file Table_1.DOCX]

**Table S1. Phenotypic variation and genetic analysis of 21 traits using 175 F_9:11_ soybean RILs under conditions with rhizobial inoculation in the ﬁeld.**

| Traits | Parents | | RILs | | | | | | | |
| --- | --- | --- | --- | --- | --- | --- | --- | --- | --- | --- |
|  | JD12 | NF58 | Max | Min | Mean | SD | CV/% | Kurt | Skew | *h^2^_b_* |
| NTN | 74.67 | 46.33 | 438.33 | 2.89 | 76.54 | 59.71 | 78.01 | 8.17 | 2.16 | 0.9 |
| WTN | 0.48 | 0.28 | 0.96 | 0.01 | 0.28 | 0.19 | 69.07 | 1.11 | 1.06 | 0.87 |
| NBN | 68.75 | 34.89 | 209.44 | 1.22 | 46.67 | 35.06 | 75.13 | 3.42 | 1.54 | 0.89 |
| WBN | 0.52 | 0.26 | 0.9 | 0.01 | 0.25 | 0.18 | 70.55 | 1.24 | 1.08 | 0.86 |
| NSN | 13.56 | 11.44 | 268 | 0.86 | 29.87 | 31.08 | 104.04 | 22.04 | 3.74 | 0.88 |
| WSN | 0.02 | 0.01 | 0.27 | 0 | 0.03 | 0.03 | 100.5 | 19.93 | 3.48 | 0.84 |
| RDW | 4.44 | 3.42 | 7.11 | 1.42 | 3.82 | 1.1 | 28.79 | -0.37 | 0.23 | 0.88 |
| SDW | 89.53 | 55.27 | 216.64 | 27.61 | 63.57 | 19.15 | 30.13 | 22.43 | 2.89 | 0.57 |
| TRL | 774.23 | 808.5 | 3909.47 | 362.65 | 1685.11 | 691.07 | 41.01 | 0.53 | 0.81 | 0.63 |
| FRL | 647.89 | 685.76 | 3553.68 | 313.93 | 1508.76 | 625.15 | 41.43 | 0.52 | 0.79 | 0.61 |
| MRL | 99.31 | 85.2 | 299.28 | 32.53 | 127.51 | 53.92 | 42.29 | 0.68 | 0.84 | 0.69 |
| CRL | 25.85 | 36.64 | 121.48 | 11.23 | 44.39 | 22.83 | 51.43 | 1.25 | 1.25 | 0.72 |
| TRSA | 177.33 | 181.52 | 646.32 | 87.88 | 278.91 | 111.51 | 39.98 | 1.01 | 1.05 | 0.7 |
| FRSA | 67.83 | 72.68 | 328.14 | 38.13 | 134.37 | 56.05 | 41.71 | 1.45 | 1.09 | 0.64 |
| MRSA | 42.09 | 36.58 | 128.48 | 13.34 | 54.63 | 23.61 | 43.21 | 0.63 | 0.84 | 0.69 |
| CRSA | 52.92 | 54.46 | 157.12 | 16.98 | 67.87 | 29.43 | 43.36 | 0.71 | 1 | 0.71 |
| TRV | 16.21 | 12.77 | 41.97 | 3.91 | 18.18 | 8.06 | 44.34 | 0.1 | 0.81 | 0.69 |
| FRV | 0.87 | 0.88 | 3.9 | 0.48 | 1.51 | 0.63 | 41.9 | 2.33 | 1.32 | 0.65 |
| MRV | 1.47 | 1.3 | 4.63 | 0.44 | 1.93 | 0.85 | 44.2 | 0.58 | 0.83 | 0.69 |
| CRV | 13.86 | 10.59 | 36.74 | 2.55 | 14.74 | 6.96 | 47.22 | 0.14 | 0.82 | 0.65 |
| ARD | 0.75 | 0.71 | 0.91 | 0.43 | 0.56 | 0.09 | 15.48 | 2.19 | 1.34 | 0.46 |

Note: roots were separated into 3 groups based on the root diameter (RD), including fine (RD < 1mm), medium roots (1mm≤RD < 2mm) and coarse roots (RD≥ 2mm). 21 traits included six BNF (biological nitrogen fixation) traits: NTN (number of total nodule, #/plant), WTN (weight of total nodule, g/plant), NBN (number of big nodules, #/plant), WBN (dry weight of big nodules, g/plant), NSN (number of small nodules, #/plant) and WSN (dry weight of small nodules, g/plant); 14 RA (root architecture) traits: TRL (total root length, cm/plant), FRL (fine root length, cm/plant), MRL (medium root length, cm/plant), CRL (coarse root length, cm/plant), TRSA (total root surface area, cm2/plant), FRSA (surface area of fine roots, cm2/plant), MRSA (surface area of medium roots, cm2/plant), CRSA (surface area of coarse roots, cm2/plant), TRV (total root volume, cm3/plant), FRV (fine root volume, cm3/plant), MRV (medium root volume, cm3/plant), CRV (coarse root volume, cm3/plant), ARD (average root diameter, mm) and RDW (root dry weight, g/plant); and SDW (shoot dry weight g/plant).
