# Supplementary material for: Characterization of Genetic Basis on Synergistic Interactions between Root Architecture and Biological Nitrogen Fixation in Soybean
Source: Front Plant Sci. 2017 Aug 23;8:1466. doi: 10.3389/fpls.2017.01466 (PMC5572596; doi:10.3389/fpls.2017.01466)
Supplement: Supplementary file 2 [file Table_2.DOCX]

**Table S2. Pearson correlation coefﬁcients among 21 traits using 175 F_9:11_ soybean RILs in the ﬁeld.**

| Trait | NTN | WTN | NBN | WBN | NSN | WSN | RDW | SDW | TRL | FRL | MRL | CRL | TRSA | FRSA | MRSA | CRSA | ARD | TRV | FRV | MRV | CRV |
| --- | --- | --- | --- | --- | --- | --- | --- | --- | --- | --- | --- | --- | --- | --- | --- | --- | --- | --- | --- | --- | --- |
| NTN |  | 0.8537*** | 0.9141*** | 0.7807*** | 0.8888*** | 0.8867*** | 0.6441*** | 0.4062*** | 0.4903*** | 0.4794*** | 0.4938*** | 0.5129*** | 0.5247*** | 0.4764*** | 0.4955*** | 0.5056*** | 0.1219ns | 0.4964*** | 0.4705*** | 0.4969*** | 0.4712*** |
| WTN | 0.8562*** |  | 0.9464*** | 0.9894*** | 0.5710*** | 0.6249*** | 0.6790*** | 0.5223*** | 0.4666*** | 0.4572*** | 0.4736*** | 0.4531*** | 0.4966*** | 0.4540*** | 0.4756*** | 0.4586*** | 0.0952ns | 0.4742*** | 0.4464*** | 0.4768*** | 0.4502*** |
| NBN | 0.8828*** | 0.9509*** |  | 0.9242*** | 0.6269*** | 0.6621*** | 0.6857*** | 0.4765*** | 0.5085*** | 0.4991*** | 0.5040*** | 0.4957*** | 0.5374*** | 0.4944*** | 0.5051*** | 0.4952*** | 0.1080ns | 0.5019*** | 0.4862*** | 0.5057*** | 0.4750*** |
| WBN | 0.7407*** | 0.9777*** | 0.9239*** |  | 0.4562*** | 0.5057*** | 0.6654*** | 0.5292*** | 0.4487*** | 0.4400*** | 0.4550*** | 0.4280*** | 0.4771*** | 0.4371*** | 0.4567*** | 0.4368*** | 0.0906ns | 0.4579*** | 0.4296*** | 0.4578*** | 0.4351*** |
| NSN | 0.9015*** | 0.5917*** | 0.5928*** | 0.4187*** |  | 0.9558*** | 0.4636*** | 0.2427** | 0.3701*** | 0.3597*** | 0.3806*** | 0.4257*** | 0.4028*** | 0.3589*** | 0.3827*** | 0.4124*** | 0.1113ns | 0.3874*** | 0.3562*** | 0.3847*** | 0.3692*** |
| WSN | 0.8972*** | 0.6263*** | 0.6189*** | 0.4511*** | 0.9687*** |  | 0.4669*** | 0.2621*** | 0.3640*** | 0.3549*** | 0.3700*** | 0.3971*** | 0.3903*** | 0.3514*** | 0.3723*** | 0.3823*** | 0.0864ns | 0.3609*** | 0.3461*** | 0.3744*** | 0.3406*** |
| RDW | 0.5679*** | 0.6135*** | 0.6225*** | 0.5976*** | 0.4010*** | 0.3983*** |  | 0.7164*** | 0.6152*** | 0.5918*** | 0.6934*** | 0.7361*** | 0.6972*** | 0.5907*** | 0.6997*** | 0.7410*** | 0.3175*** | 0.7296*** | 0.5850*** | 0.7049*** | 0.7054*** |
| SDW | 0.4686*** | 0.5598*** | 0.5385*** | 0.5646*** | 0.3091*** | 0.2947*** | 0.8140*** |  | 0.4016*** | 0.3932*** | 0.3967*** | 0.4247*** | 0.4322*** | 0.3768*** | 0.3992*** | 0.4456*** | 0.1635* | 0.4508*** | 0.3616*** | 0.4011*** | 0.4400*** |
| TRL | 0.3780*** | 0.3693*** | 0.3755*** | 0.3391*** | 0.3025*** | 0.2998*** | 0.3941*** | 0.2340** |  | 0.9983*** | 0.8859*** | 0.7591*** | 0.9689*** | 0.9896*** | 0.8793*** | 0.7099*** | -0.0684ns | 0.7059*** | 0.9693*** | 0.8720*** | 0.6224*** |
| FRL | 0.3596*** | 0.3548*** | 0.3590*** | 0.3265*** | 0.2862*** | 0.2841*** | 0.3620*** | 0.2076** | 0.9977*** |  | 0.8580*** | 0.7236*** | 0.9540*** | 0.9877*** | 0.8505*** | 0.6751*** | -0.1040ns | 0.6747*** | 0.9640*** | 0.8422*** | 0.5903*** |
| MRL | 0.4470*** | 0.4063*** | 0.4268*** | 0.3669*** | 0.3739*** | 0.3645*** | 0.5931*** | 0.4318*** | 0.7142*** | 0.6659*** |  | 0.8923*** | 0.9533*** | 0.8819*** | 0.9993*** | 0.8408*** | 0.1966** | 0.8219*** | 0.8941*** | 0.9974*** | 0.7482*** |
| CRL | 0.3783*** | 0.3155*** | 0.3517*** | 0.2768*** | 0.3267*** | 0.3188*** | 0.5383*** | 0.3868*** | 0.4517*** | 0.3967*** | 0.8066*** |  | 0.8758*** | 0.7459*** | 0.9022*** | 0.9639*** | 0.3362*** | 0.8980*** | 0.7560*** | 0.9109*** | 0.8596*** |
| TRSA | 0.4635*** | 0.4226*** | 0.4488*** | 0.3823*** | 0.3825*** | 0.3755*** | 0.5234*** | 0.3523*** | 0.8917*** | 0.8626*** | 0.9098*** | 0.7249*** |  | 0.9699*** | 0.9500*** | 0.8319*** | 0.1088ns | 0.8150*** | 0.9691*** | 0.9457*** | 0.7398*** |
| FRSA | 0.3908*** | 0.3738*** | 0.3905*** | 0.3432*** | 0.3110*** | 0.3069*** | 0.3955*** | 0.2412** | 0.9688*** | 0.9603*** | 0.7635*** | 0.4927*** | 0.9417*** |  | 0.8729*** | 0.6947*** | -0.0316ns | 0.6908*** | 0.9933*** | 0.8632*** | 0.6037*** |
| MRSA | 0.4511*** | 0.4090*** | 0.4283*** | 0.3685*** | 0.3796*** | 0.3706*** | 0.6001*** | 0.4355*** | 0.7052*** | 0.6561*** | 0.9990*** | 0.8227*** | 0.9043*** | 0.7503*** |  | 0.8506*** | 0.2045** | 0.8292*** | 0.8839*** | 0.9994*** | 0.7573*** |
| CRSA | 0.3295*** | 0.3021*** | 0.3262*** | 0.2780*** | 0.2669*** | 0.2636*** | 0.5524*** | 0.4269*** | 0.3972*** | 0.3468*** | 0.7192*** | 0.9356*** | 0.6546*** | 0.4324*** | 0.7335*** |  | 0.3470*** | 0.9756*** | 0.7047*** | 0.8594*** | 0.9606*** |
| ARD | -0.0032ns | -0.0490ns | -0.0248ns | -0.0539ns | 0.0190ns | 0.0169ns | 0.1129ns | 0.1495* | -0.5630*** | -0.6047*** | 0.0309ns | 0.2583*** | -0.1875* | -0.4450*** | 0.0426ns | 0.2646*** |  | 0.3079*** | 0.0217ns | 0.2119** | 0.3288*** |
| TRV | 0.3662*** | 0.3520*** | 0.3728*** | 0.3290*** | 0.2865*** | 0.2883*** | 0.5991*** | 0.4678*** | 0.4803*** | 0.4370*** | 0.7245*** | 0.8308*** | 0.6939*** | 0.5104*** | 0.7333*** | 0.9545*** | 0.1717* |  | 0.6992*** | 0.8354*** | 0.9923*** |
| FRV | 0.3933*** | 0.3677*** | 0.3919*** | 0.3361*** | 0.3141*** | 0.3097*** | 0.4065*** | 0.2582*** | 0.9100*** | 0.8935*** | 0.8122*** | 0.5438*** | 0.9563*** | 0.9822*** | 0.7969*** | 0.4793*** | -0.3187*** | 0.5457*** |  | 0.8730*** | 0.6116*** |
| MRV | 0.4546*** | 0.4112*** | 0.4292*** | 0.3696*** | 0.3848*** | 0.3763*** | 0.6060*** | 0.4385*** | 0.6949*** | 0.6450*** | 0.9958*** | 0.8376*** | 0.8974*** | 0.7359*** | 0.9990*** | 0.7467*** | 0.0544ns | 0.7410*** | 0.7806*** |  | 0.7655*** |
| CRV | 0.3158*** | 0.3080*** | 0.3269*** | 0.2907*** | 0.2424** | 0.2461*** | 0.5604*** | 0.4487*** | 0.3587*** | 0.3176*** | 0.6079*** | 0.7797*** | 0.5708*** | 0.3798*** | 0.6192*** | 0.9406*** | 0.2233** | 0.9863*** | 0.4123*** | 0.6295*** |  |

Note: Pearson correlation coefﬁcients under without (-) or with (+) rhizobial inoculation conditions are listed below or up the diagonal. Statistically significant relationships are indicated by * (p< 0.05), ** (p< 0.01) and *** (p< 0.001). ns: not significant.
