# Supplementary figures and images for: Characterization of Genetic Basis on Synergistic Interactions between Root Architecture and Biological Nitrogen Fixation in Soybean
Source: Front Plant Sci. 2017 Aug 23;8:1466. doi: 10.3389/fpls.2017.01466 (PMC5572596; doi:10.3389/fpls.2017.01466)

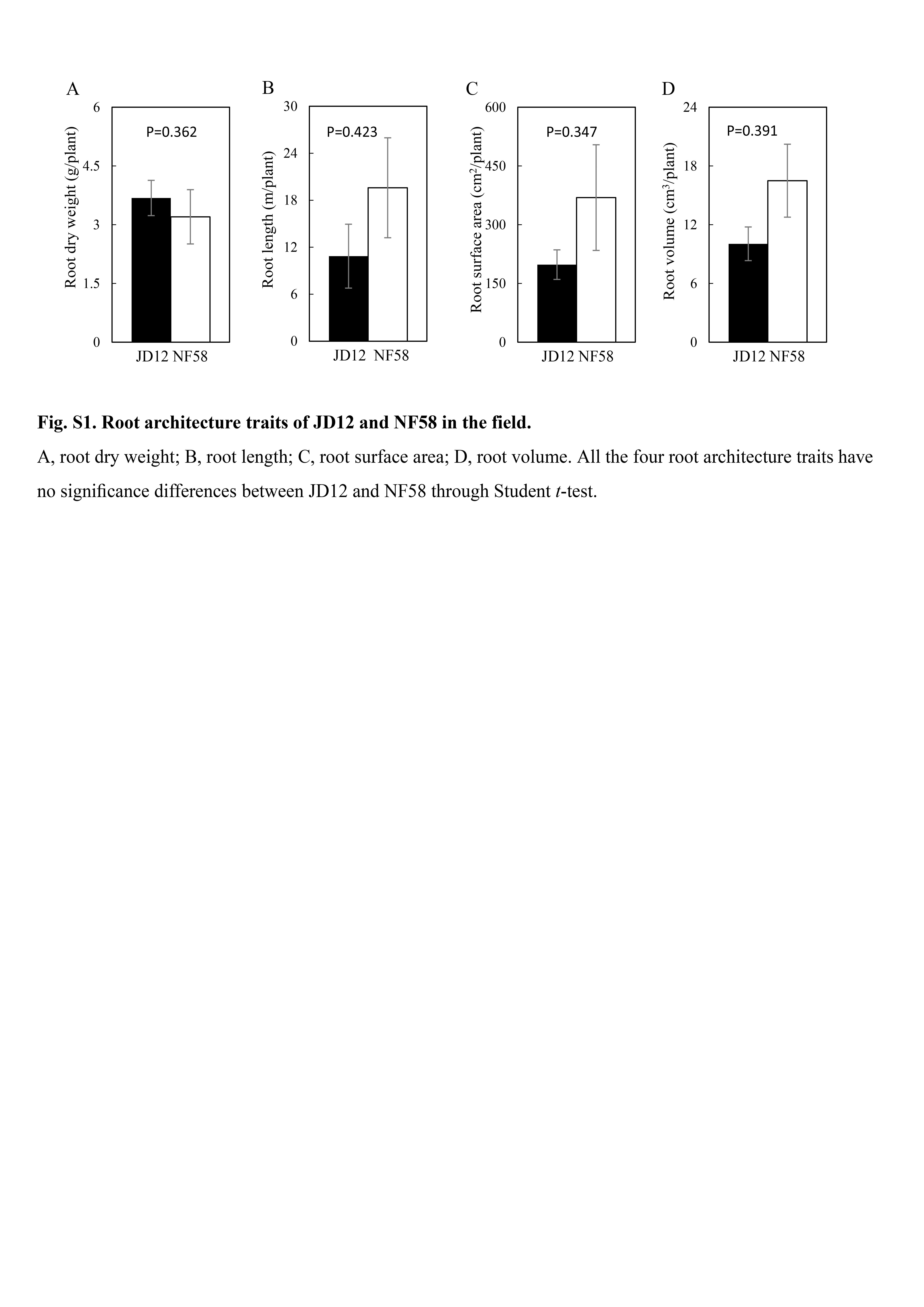

Supplement: Supplementary file 3 [file Image_1.TIF]

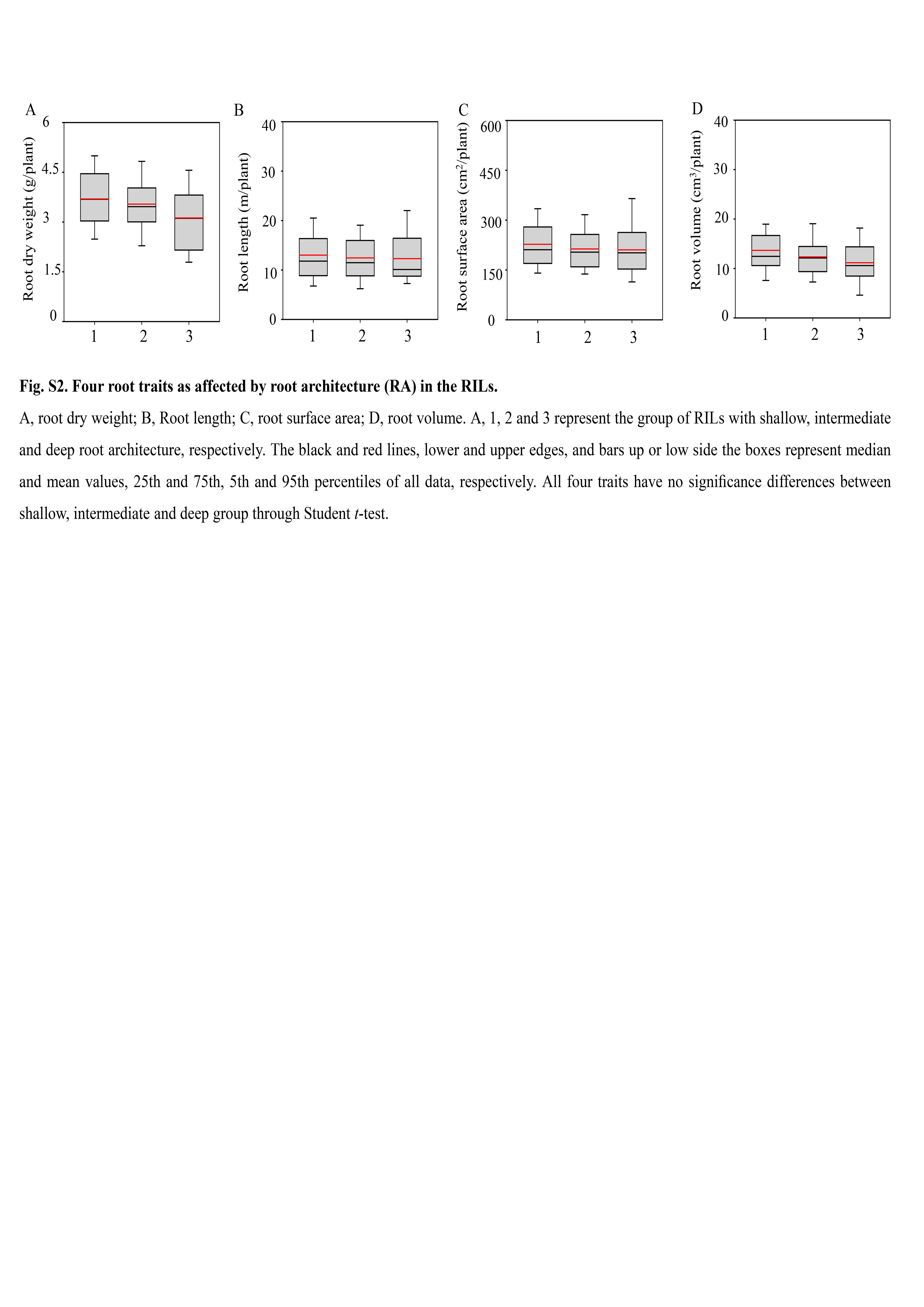

Supplement: Supplementary file 4 [file Image_2.TIF]

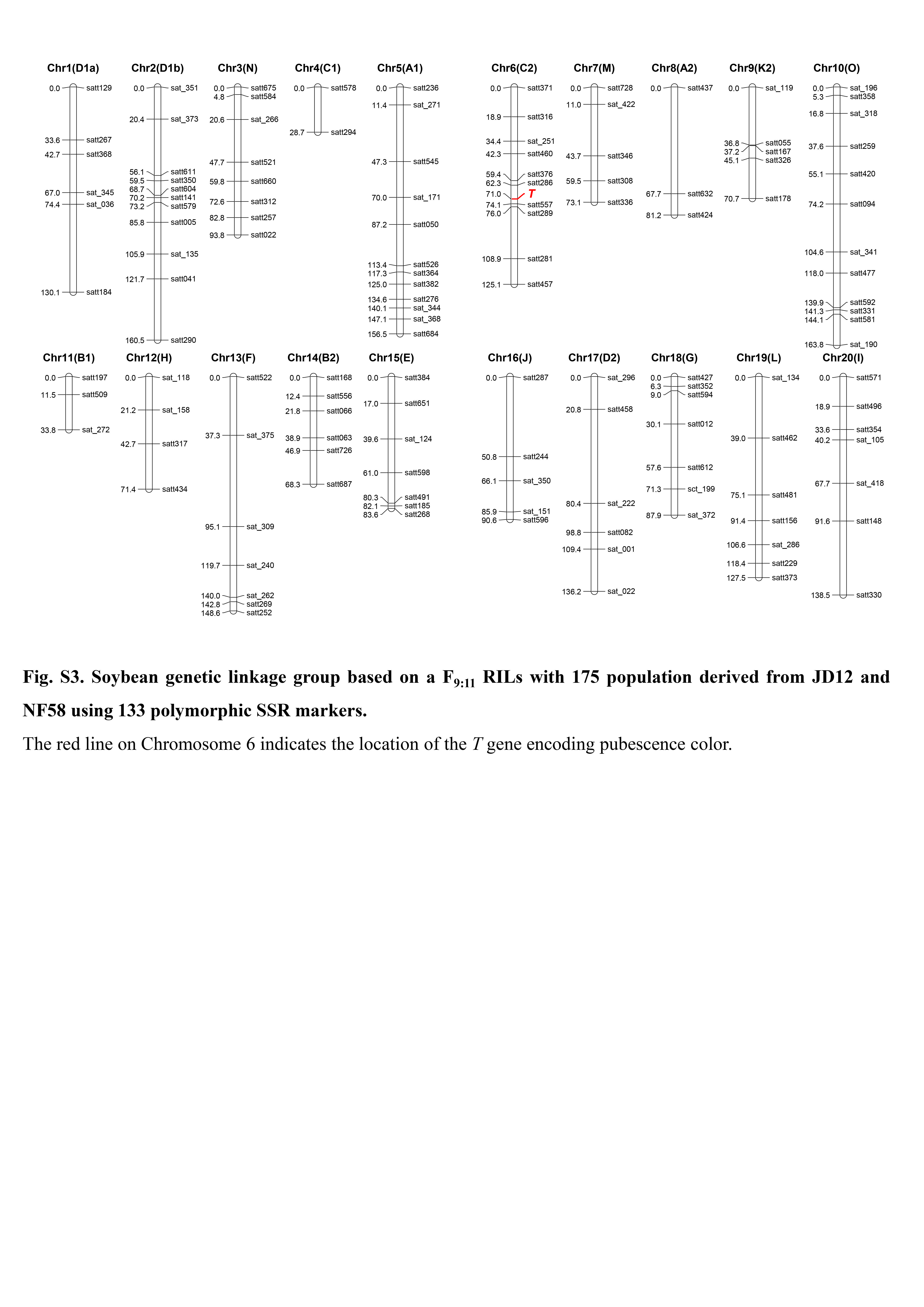

Supplement: Supplementary file 5 [file Image_3.TIF]

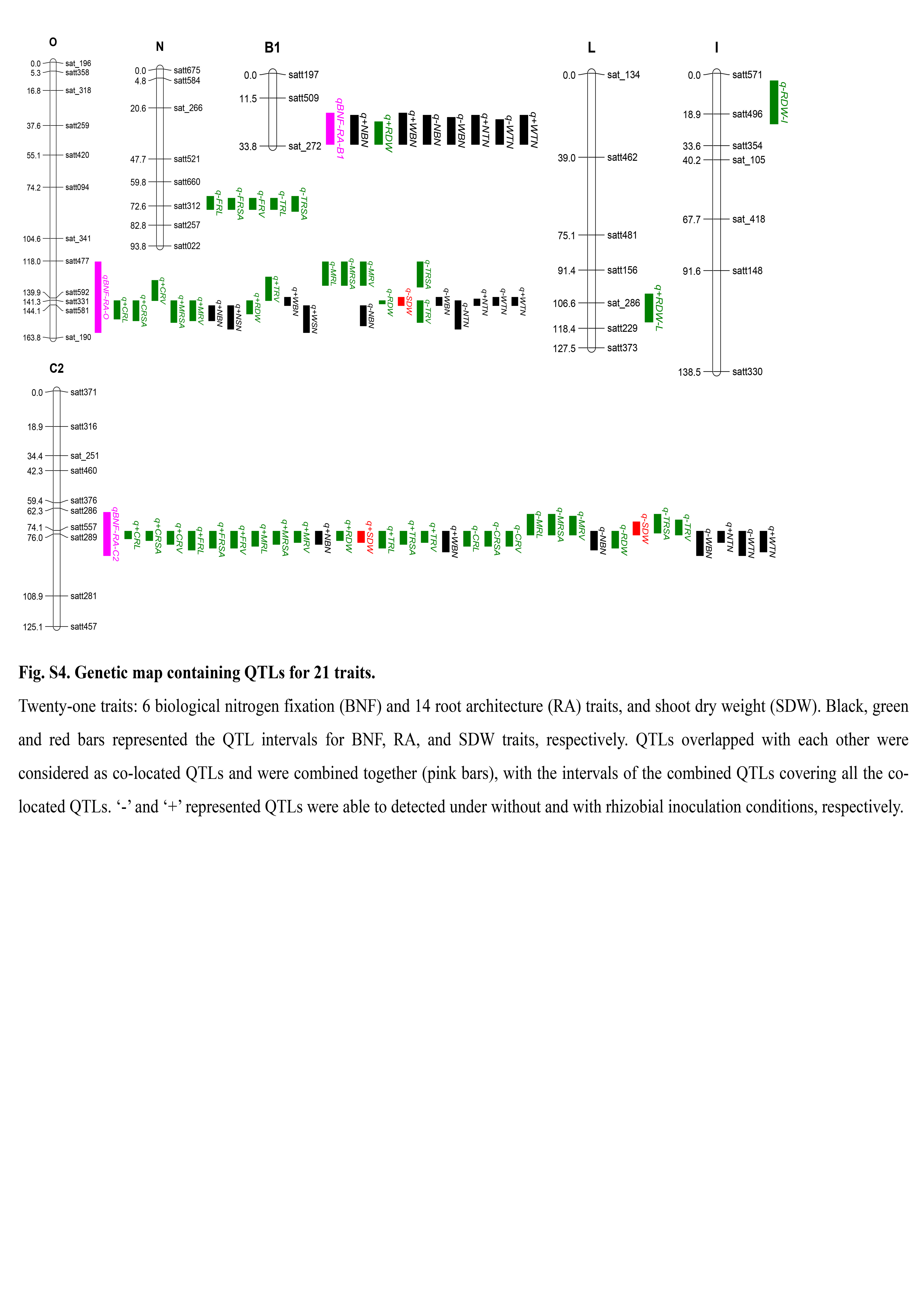

Supplement: Supplementary file 6 [file Image_4.TIF]
